# Beyond Blood Pressure: Salt Sensitivity as a Cardiorenal Phenotype—A Narrative Review

**DOI:** 10.3390/life16020247

**Published:** 2026-02-02

**Authors:** Maria Bachlitzanaki, Georgios Aletras, Eirini Bachlitzanaki, Nektaria Vasilaki, Charalampos Lydakis, Ioannis Petrakis, Emmanuel Foukarakis, Kostas Stylianou

**Affiliations:** 1 Department of Internal Medicine, Venizelio General Hospital of Heraklion, 71409 Heraklion, Greece; mariabachlitzanaki@gmail.com (M.B.); vasilakinektaria@hotmail.com (N.V.); hlydakis@yahoo.gr (C.L.); 2 Department of Cardiology, Venizelio General Hospital of Heraklion, 71409 Heraklion, Greece; medp2012222@med.uoc.gr (G.A.); eirinibach@outlook.com.gr (E.B.); mfouk@hotmail.com (E.F.); 3 School of Medicine, University of Crete, 71003 Heraklion, Greece; petrakgia@uoc.gr; 4 Department of Nephrology, University General Hospital of Heraklion, 71500 Heraklion, Greece

**Keywords:** salt-sensitive blood pressure, sodium, renin–angiotensin–aldosterone system, heart failure, cardiorenal syndrome

## Abstract

**Background:** Salt-sensitive blood pressure (SSBP) represents a prevalent yet underrecognized hypertensive phenotype, in which blood pressure (BP) and volume status are disproportionately influenced by dietary sodium intake. Beyond BP elevation alone, salt sensitivity reflects a convergence of renal sodium handling abnormalities, neurohormonal activation, vascular dysfunction, and inflammatory pathways that link excessive sodium exposure to progressive kidney injury and adverse cardiac remodeling. Given its association with chronic kidney disease (CKD) and the association of heart failure with preserved ejection fraction (HFpEF), improved recognition of SSBP has direct clinical relevance. **Objective:** This narrative review aims to synthesize current mechanistic and clinical evidence on SSBP, focusing on pathophysiology, cardiorenal interactions, diagnostic challenges, and phenotype-guided therapeutic strategies with practical applicability. **Methods:** A narrative literature review was conducted using PubMed, Scopus, and Web of Science from inception through January 2026. Experimental, translational, and clinical studies, along with relevant guideline documents, were integrated to provide conceptual and clinical interpretation rather than quantitative analysis. **Key Findings:** Impaired renal sodium excretion, intrarenal RAAS activation, sympathetic overactivity, endothelial dysfunction, and immune-mediated inflammation contribute to sodium retention, microvascular dysfunction, and fibrotic remodeling across the kidney–heart axis. These pathways are strongly supported by experimental and translational data, but direct interventional clinical validation remains limited for several mechanisms. Clinically, salt-sensitive individuals often exhibit non-dipping BP patterns, albuminuria, salt-induced edema, and a predisposition to HFpEF. Dynamic BP monitoring combined with targeted laboratory assessment improves identification of this phenotype and supports individualized management. **Conclusions:** Early recognition of SSBP enables targeted interventions beyond uniform sodium restriction. Phenotype-guided strategies integrating lifestyle modification, RAAS blockade, thiazide-like diuretics, mineralocorticoid receptor antagonists, and sodium-glucose co-transporters 2 inhibitors (SGLT2i) may improve cardiorenal outcomes. Emerging precision tools (e.g., wearable blood-pressure sensors, digital sodium tracking technologies, etc.) remain exploratory but may further refine individualized management.

## 1. Introduction

Sodium chloride, commonly known as salt, is an essential nutrient with vital physiological and hemodynamic functions. It has a fundamental role in maintaining extracellular fluid volume, osmotic balance, and vascular tone, while it is crucial for neuromuscular excitability, membrane potential regulation, and nutrient transport across cell membranes via sodium-dependent cotransporters [1]. In the context of the salt–kidney axis, sodium also modulates renal perfusion, glomerular filtration, and tubular function through its effects on the renin–angiotensin–aldosterone system (RAAS) and the sympathetic nervous system, as demonstrated primarily in experimental and translational models with supportive clinical observations [2].

Under normal conditions, the body adapts to variations in salt intake to maintain circulatory homeostasis. However, excessive sodium restriction can disrupt this balance by triggering compensatory mechanisms such as RAAS and sympathetic activation, leading to vasoconstriction, endothelial dysfunction, and insulin resistance. This paradox is particularly relevant in patients with cardiovascular disease, where aggressive sodium restriction may worsen neurohormonal activation and renal perfusion [3]. Indeed, daily sodium intake below 1.5 g/day has been associated with increased hospitalization and mortality, most likely due to activation of neurohormonal and inflammatory pathways [3,4].

Conversely, excessive dietary sodium intake is closely linked to blood pressure (BP) elevation, and is a well-established contributor to cardiovascular events and target organ damage [5,6]. Despite longstanding public health recommendations advocating sodium restriction, average daily intake remains above the recommended levels in most populations regardless of age, sex or regional epidemiological profile [4]. Within this context, salt-sensitive blood pressure (SSBP) has emerged as a clinically relevant yet underrecognized hypertensive phenotype, in which BP and volume status are disproportionately influenced by dietary sodium intake. Importantly, salt sensitivity carries prognostic implications that extend beyond office BP values, linking sodium handling to cardiorenal injury and heart failure (HF) phenotypes. However, assessment of salt sensitivity remains limited in routine clinical practice, and its implications for contemporary cardiovascular and renal care are often overlooked [7,8].

Sodium restriction remains a key strategy in hypertension management; however, the optimal intake should achieve a careful balance—low enough to prevent volume overload, especially in HF, but not so low as to provoke adverse neurohormonal responses [1,3,9].

Accordingly, this narrative review aims to synthesize current evidence on salt-sensitive hypertension, explicitly distinguishing mechanistic insights from clinically validated data, focusing on its pathophysiological basis, cardiorenal interactions, diagnostic challenges, and phenotype-guided therapeutic strategies, with particular emphasis on clinical applicability.

## 2. Objectives

SSBP represents a clinically relevant but inconsistently recognized phenotype at the intersection of hypertension, chronic kidney disease (CKD) and HF. Despite extensive experimental and clinical literature, evidence remains fragmented across disciplines, limiting translation into routine clinical practice.

The primary objective of this narrative review is to conceptually integrate current experimental, translational, and clinical evidence describing salt sensitivity as a unifying cardiorenal phenotype.

Secondary objectives are:

1. To summarize key pathophysiological mechanisms linking sodium handling to renal and cardiovascular injury, emphasizing convergent pathways rather than exhaustive molecular detail.
2. To highlight clinically recognizable manifestations of SSBP across renal and cardiac phenotypes.
3. To discuss practical diagnostic and monitoring approaches applicable to real-world clinical settings.
4. To outline phenotype-oriented therapeutic principles supporting individualized mechanism-informed management rather than uniform blood pressure targets.

This review is intended to support clinical reasoning and translational understanding, rather than to provide quantitative effect estimates or formal guideline recommendations.

## 3. Literature Review Strategy

This manuscript is a narrative review designed to provide a conceptual and integrative synthesis of current knowledge on SSBP and its cardiorenal implications. The intent was interpretative rather than exhaustive, prioritizing mechanistic coherence and clinical relevance over formal evidence grading.

A targeted literature search was conducted using PubMed, Scopus, and Web of Science, covering publications from database inception through January 2026. Search terms included combinations of *“salt sensitivity,” “salt-sensitive blood pressure,” “salt-sensitive hypertension,” “sodium intake,” “renal sodium handling,” “RAAS,” “mineralocorticoid receptor,” “HFpEF,” “chronic kidney disease,” “inflammation,” “fibrosis,”* and *“cardiorenal syndrome.”* Only English-language articles were considered.

Studies were selected based on relevance to the conceptual framework of salt sensitivity, including:

▪ Experimental and translational studies elucidating mechanisms of sodium handling and organ injury;
▪ Observational and interventional clinical studies relevant to hypertension, CKD, and HFpEF;
▪ Contemporary guideline statements and authoritative reviews from major scientific societies.

Consistent with the narrative design, no predefined inclusion or exclusion criteria, PRISMA flow diagram, quantitative synthesis, or formal risk-of-bias assessment was applied. Differences in study design and level of evidence are explicitly acknowledged within the text where relevant.

## 4. Salt-Sensitive Hypertension: The Forgotten Player in the Hypertension Playground?

Contrary to common belief, not every individual who consumes a high-salt diet develops elevated BP. The response to dietary sodium varies significantly across individuals and populations, a phenomenon known as salt sensitivity. Individuals who are salt-sensitive are more prone to developing hypertension in response to sodium intake, whereas salt-resistant individuals maintain stable BP despite changes in sodium consumption [10,11].

### 4.1. Definition, Epidemiology, and Conceptual Framework of Salt Sensitivity

Salt-sensitive hypertension represents a distinct and clinically relevant phenotype of hypertension, characterized by exaggerated BP response to changes in dietary sodium intake [6,12]. Epidemiological data suggest that SSBP affects nearly 50% of patients with hypertension and approximately 25% of those who are normotensive, highlighting its significance as an underrecognized public health concern [10]. Although sodium restriction has proven effective in lowering BP among salt-sensitive individuals, the widespread presence of “hidden” salt in processed and ultra-processed foods—combined with structural inequities in food systems—has shifted salt intake from a conscious to a largely passive behavior. This complicates population-level interventions and may limit the efficacy of dietary reduction efforts [5].

**Figure 1:**
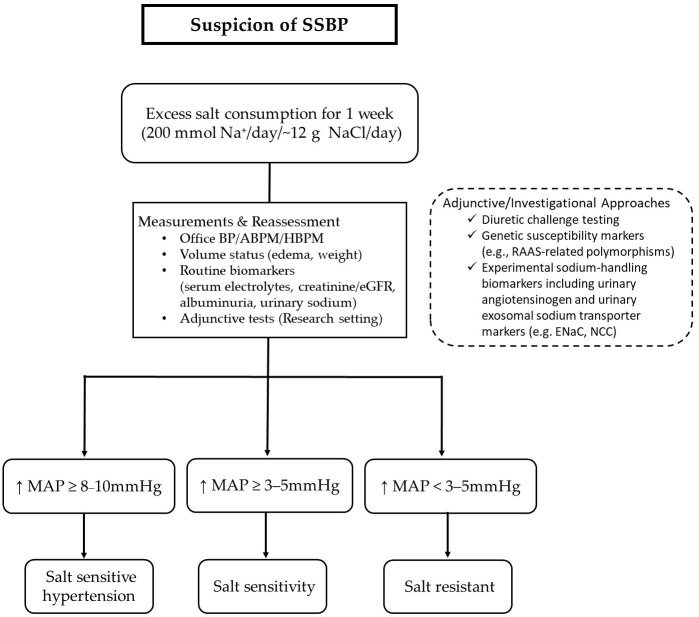
Conceptual framework for the assessment of salt sensitivity. Salt sensitivity is traditionally evaluated by observing BP responses to controlled dietary sodium manipulation, typically involving sequential low-salt and high-salt intake over several days to one week, with standardized BP measurements. Assessment relies on dynamic BP evaluation using office measurements, ABPM, or HBPM, in combination with reassessment of volume status and routine biomarkers of sodium balance and renal function, including serum electrolytes, creatinine/eGFR, albuminuria, and urinary sodium excretion. Adjunctive and investigational approaches are shown separately to emphasize their supportive and mechanistic role. These include diuretic challenge testing, genetic susceptibility markers (e.g., RAAS-related polymorphisms), and experimental sodium-handling biomarkers such as urinary angiotensinogen and urinary exosomal sodium transporter markers (e.g., ENaC, NCC). These methods are primarily applied in research or selected clinical settings and are not currently standardized for routine clinical diagnosis of salt sensitivity. Given the absence of a universally accepted clinical gold standard, the framework illustrates commonly used criteria and approaches in clinical and translational studies rather than a definitive diagnostic algorithm. Abbreviations: ABPM, ambulatory blood pressure monitoring; BP, blood pressure; eGFR, estimated glomerular filtration rate; ENaC, epithelial sodium channel; HBPM, home blood pressure monitoring; MAP, mean arterial pressure; NCC, sodium–chloride cotransporter; RAAS, renin–angiotensin–aldosterone system.

The reference method for assessing salt sensitivity involves sequential dietary phases—typically one week of high-salt intake followed by one week of low-salt intake—accompanied by standardized BP measurements. The most widely accepted cut-off points define salt sensitivity as an increase in mean arterial pressure (MAP) of ≥3–5 mmHg in normotensive individuals, whereas an increase of ≥8–10 mmHg defines salt-sensitive hypertension. Individuals with blood pressure changes <3–5 mmHg are considered salt-resistant [2]. The proposed algorithm for the assessment of salt sensitivity is depicted in Figure 1.

Salt sensitivity is influenced by a combination of genetic, developmental, and acquired factors. These include older age, female sex, obesity, low potassium intake, and African ancestry. Developmental factors such as low birth weight are particularly relevant, as they are linked to reduced nephron number (oligonephronia), limiting the kidney’s lifelong capacity to excrete sodium efficiently. Furthermore, individuals with a low-renin phenotype—often reflecting heightened sodium sensing at the juxtaglomerular apparatus—show an exaggerated suppression of renin in response to salt intake, weakening compensatory natriuretic mechanisms and predisposing to SSBP elevations. Metabolic disturbances, especially insulin resistance, further amplify this effect: insulin normally stimulates sodium reabsorption in distal nephron segments rich in epithelial sodium channels (ENaC), and hyperinsulinemia enhances this sodium-retaining action. Unsurprisingly, SSBP is more common among individuals with hypertension, CKD, and cardiometabolic disease, where impaired natriuresis, sympathetic overactivity, and renal microvascular dysfunction converge to promote a salt-sensitive phenotype [13,14,15,16,17].

### 4.2. Limitations and Emerging Approaches in the Diagnosis of Salt Sensitivity

Despite growing recognition of SSBP as a clinically relevant phenotype, its diagnosis remains poorly standardized, and no universally accepted “gold standard” exists. Traditional diagnostic protocols based on sequential dietary sodium loading and depletion are primarily used in research settings and face several important limitations that restrict their applicability in routine clinical practice [4,18].

These protocols are labor-intensive, require strict dietary adherence, and are highly sensitive to confounding factors such as baseline blood pressure variability, concomitant medications, renal function, and neurohormonal status [18]. Moreover, the duration and magnitude of sodium manipulation vary substantially across studies, resulting in heterogeneous diagnostic thresholds and limited reproducibility [11]. These protocols also provide only a short-term hemodynamic snapshot and may fail to capture chronic salt sensitivity patterns relevant to long-term cardiorenal risk [18,19].

In response to these limitations, alternative diagnostic approaches have been proposed. ABPM has emerged as a practical tool to identify salt-sensitive phenotypes indirectly, particularly through detection of non-dipping or nocturnal hypertension, increased blood pressure variability, and exaggerated morning surges—patterns frequently associated with impaired sodium handling [18,20,21]. However, ABPM does not directly quantify salt sensitivity and remains a surrogate marker rather than a definitive diagnostic test [11,18].

Biomarker-based approaches, including urinary sodium excretion, aldosterone-to-renin ratio, albuminuria, and emerging renal injury markers (e.g., NGAL, cystatin C), may provide supportive evidence of sodium-driven pathophysiology [22,23]. Nevertheless, these markers lack specificity, are influenced by dietary and pharmacological factors, and have not been validated as standalone diagnostic tools for salt sensitivity [4,18].

Functional testing strategies, such as diuretic challenge tests or assessment of natriuretic response to dietary sodium modification, offer mechanistic insight but remain insufficiently standardized and are rarely implemented outside specialized centers [24,25].

Collectively, current diagnostic strategies highlight a critical gap between mechanistic understanding and clinical implementation. Rather than a single diagnostic test, salt sensitivity is best conceptualized as a phenotype identified through integration of hemodynamic patterns, laboratory markers, and clinical context [2,26]. Future efforts should focus on validating pragmatic diagnostic frameworks that combine dynamic blood pressure monitoring with mechanistically informed biomarkers to improve early identification and risk stratification in routine practice [27,28].

## 5. Pathophysiological Basis

Despite numerous studies over the past 50 years, the pathophysiology behind SSBP response remains incompletely understood [13]. The prevailing concept is that individuals with normal sodium regulation can efficiently excrete excess sodium through renal or extrarenal mechanisms, maintaining stable BP. In contrast, salt-sensitive individuals exhibit impaired sodium excretion, leading to sodium retention, plasma volume expansion, and BP elevation. This variability in hemodynamic response defines the salt sensitivity spectrum. To better understand this heterogeneous condition, multiple mechanisms and genetic contributors have been explored, ranging from experimental and translational models to observational human studies, while direct interventional clinical validation remains limited for several pathways [2].

### 5.1. Renal Mechanisms

The kidney plays a central role in SSBP, a concept supported by robust experimental data and consistent observational evidence in human hypertension and CKD cohorts, primarily due its inability to excrete sodium appropriately in response to increased dietary intake. This results in sodium retention and intravascular volume overload, triggering blood pressure elevation [29].

The renin–angiotensin–aldosterone system is a critical regulator of blood volume, electrolyte balance, and systemic vascular resistance, mediating both acute and chronic physiological adaptations [30]. Its three key effectors—renin, angiotensin II, and aldosterone—act in concert to raise arterial BP in response to reduced renal perfusion, decreased sodium delivery to the distal tubule, or sympathetic stimulation via β-adrenergic receptors. RAAS exerts its influence across multiple organ systems—including the kidneys, vasculature, lungs, adrenal glands, and central nervous system—making it a central player in cardiovascular and renal homeostasis. By modulating vascular tone, sodium retention, and water balance, the RAAS plays a pivotal role in the development of hypertension, HF, and various cardiorenal disorders. Consequently, therapeutic inhibition of RAAS overactivation remains a cornerstone in the treatment of these conditions, offering proven clinical benefit [30,31].

Beyond classical RAAS, other renal pathways have emerged as modulators of salt sensitivity. One involves “With No lysine [K] 4” (WNK4) kinase, which enhances activity of the thiazide-sensitive sodium chloride (NaCl) cotransporter (NCC) in the distal convoluted tubule. Mutations in WNK4 are linked to familial hyperkalemic hypertension due to hyperactivation of the SPAK/OSR1–NCC axis, promoting sodium reabsorption and impaired potassium secretion [32].

Another key player is Rac1, a small GTPase that modulates cytoskeletal signaling, oxidative stress, and gene expression. Under high-salt conditions, Rac-1 can aberrantly activate the mineralocorticoid receptor (MR)—even in the absence of aldosterone- through mechanisms involving inflammation, angiotensin II and mechanical stress. This promotes expression of sodium transporters, enhances sodium retention, and elevates blood pressure, providing a non-aldosterone-dependent pathway to MR activation and salt-induced hypertension. Notably, most evidence supporting Rac1-mediated MR activation derives from experimental and translational studies, while direct clinical validation in human SSBP remains limited [33].

### 5.2. Neurohumoral and Vascular Factors

The sympathetic nervous system (SNS) plays a pivotal role in BP regulation by influencing vascular tone, renal sodium handling, and cardiac output. Sympathetic overactivity is a well-established contributor to the development and maintenance of hypertension. In patients with SSBP, excess dietary sodium has been shown—primarily in experimental and physiological studies—to activate neurogenic pathways that lead to an exaggerated BP response. Evidence of sympathetic hyperactivity includes elevated norepinephrine spillover, increased muscle sympathetic nerve activity, and heightened responses to ganglionic blockade. In salt-sensitive individuals, small increases in plasma or cerebrospinal fluid sodium concentration are sensed by central regions—particularly the anteroventral third ventricle the subfornical organ and the organum vasculosum of the lamina terminalis—and initiate sympathoexcitatory responses. The final result of these signals is enhancing sympathetic drive to specific vascular beds, which acutely promotes renal sodium retention—shifting the pressure–natriuresis relationship and contributing to sustained elevations in BP [2,34].

In parallel, the vasculature itself contributes significantly to SSBP pathophysiology. Endothelial dysfunction, oxidative stress, vascular stiffening, and fibrosis are key abnormalities observed in these individuals. The endothelium plays a vital regulatory role by releasing vasodilatory factors such as nitric oxide (NO) and prostaglandins, and by exerting antioxidant, anti-inflammatory, and antithrombotic effects. However, in salt-sensitive individuals, this balance is disrupted. The endothelium shifts toward a pro-constrictive, proinflammatory, and prothrombotic phenotype, resulting in impaired vasodilation, increased vascular tone, and remodeling of resistance arteries [35,36]. These vascular changes are further amplified by RAAS activation, endothelin-1, catecholamines, and various growth factors, which together increase myogenic tone and drive both functional and structural changes in the vessel wall. The resulting vascular inflammation and remodeling contribute not only to increased peripheral resistance and sustained hypertension but also to the progression of atherosclerosis and target organ damage. While endothelial dysfunction and vascular remodeling are consistently observed in salt-sensitive states, much of the mechanistic insight originates from experimental and translational research, with clinical data largely derived from observational associations [35,36,37].

### 5.3. Immune and Inflammatory Responses

Although the precise relationship between salt intake, inflammation and cardiovascular disease continues to be explored through experimental and translational models, there is growing evidence that the immune system plays a critical role in the development of SSBP and related renal and vascular injury, with supportive but limited evidence from human observational studies [36].

Over the past two decades, increasing attention has been directed toward the contribution of innate immune activation and proinflammatory signaling in the pathogenesis of SSBP. Evidence supporting these immune-mediated mechanisms derives predominantly from experimental animal models and translational studies, with supportive—but more limited—data from human observational cohorts; direct interventional clinical evidence remains scarce [36,38].

Both innate and adaptive immune cells exhibit sodium-dependent changes in their metabolism—a field known as immunometabolism which examines how metabolic reprogramming within immune cells influences their inflammatory phenotype. In particular, excess sodium intake affects immune energy balance and hormonal regulation, particularly through the Serum and Glucocorticoid-Regulated Kinase 1 (SGK1)–Forkhead Box O1 (FOXO1) signaling axis in T helper 17 (Th17) and regulatory T (Treg) cells, thereby increasing susceptibility to autoimmune and hypertensive processes [39,40].

Among the first immune responders to sodium overload are innate antigen-presenting cells (APCs), such as macrophages and dendritic cells into the kidney, contributing to tissue injury and organ dysfunction. Elevated sodium levels also stimulate the production of reactive oxygen species (ROS) and isolevuglandin (IsoLG)-protein adducts, which activate APCs to release proinflammatory cytokines, including IL-6, IL-18, TNF-α, and IL-1β. Notably, high salt activates the NLRP3 inflammasome, further amplifying IL-1β production and perpetuating local and systemic inflammation [33,36].

Macrophage polarization also appears to play a critical role. High-salt environments favor the proinflammatory M1 phenotype, which promote cytokine release and T cell activation, whereas anti-inflammatory M2 macrophages, which secrete IL-10 and support vascular repair, are suppressed. Increased renal M1 macrophage infiltration and upregulation of markers such as IL-6, CD14, Ly96, and TLR4 have been observed in salt-loaded models, reinforcing the role of innate immunity in SSBP pathophysiology. These mechanisms have been robustly demonstrated in experimental models, whereas their direct contribution to human SSBP remains an area of active investigation [36].

Beyond classical renal and immune mechanisms, recent evidence has uncovered an extrarenal “immune–osmotic” axis that links tissue sodium accumulation with chronic inflammation. Sodium can be stored non-osmotically within the interstitium of the skin and skeletal muscle, forming a dynamic buffer compartment. Elevated interstitial sodium concentrations activate macrophages through the tonicity-responsive enhancer binding protein (TonEBP), which in turn induces vascular endothelial growth factor-C (VEGF-C)–mediated lymphangiogenesis and promotes low-grade inflammation. This immune–osmotic signaling represents a novel mechanism through which sodium excess contributes to salt sensitivity, vascular stiffness, and tissue fibrosis, extending the concept of sodium handling beyond renal excretion. Intriguingly, these findings have been confirmed not only in animal and human models but also under spaceflight conditions, highlighting their fundamental physiological relevance. Despite emerging confirmation in selected human studies, the clinical relevance and therapeutic implications of the immune–osmotic sodium storage axis remain largely exploratory [41,42].

In addition to immune cell dynamics, gut microbiota have emerged as crucial mediators linking dietary salt to systemic inflammation, vascular dysfunction, and blood pressure regulation [2]. Most available evidence for gut microbiota–mediated effects originates from experimental and translational studies, while human data remain largely observational and hypothesis-generating. High-salt diets disrupt intestinal microbial homeostasis by reducing beneficial *Lactobacillus* species and promoting dysbiosis, which increases gut permeability and systemic exposure to proinflammatory metabolites. These microbial alterations drive immune dysregulation, characterized by enhanced differentiation of proinflammatory Th17 cells and suppression of regulatory T cells, thereby amplifying vascular inflammation and oxidative stress [13,36]. These pathways contribute to endothelial injury, renal dysfunction, and BP elevation, all hallmarks of the salt-sensitive phenotype. Although the precise molecular pathways connecting gut-derived signals, immune activation, and vascular regulation remain incompletely understood, these extrarenal mechanisms are now recognized as pivotal contributors to the pathogenesis of SSBP and represent promising targets for future diagnostic and therapeutic interventions [36].

While these insights have advanced our understanding of SSBP, several challenges remain. Most notably, there is no practical diagnostic tool to readily identify salt-sensitive individuals in clinical practice [2]. Current diagnostic protocols based on salt loading and depletion remain cumbersome and limit large-scale investigations. Novel approaches incorporating immunologic and microbiome-based biomarkers may provide feasible alternatives for diagnosis and therapy in the future. A more comprehensive understanding of the interplay among the gut microbiome, immune activation, and inflammation will be essential to develop targeted strategies for preventing and managing SSBP and its cardiorenal complications [43].

### 5.4. Genetic and Epigenetic Factors

Hypertension is widely recognized as a multifactorial disease influenced by the interplay of genetic, epigenetic, and environmental factors.

It is estimated that genetic factors account for 30% to 50% of BP variability, while epigenetic modifications can influence gene expression and contribute to disease onset and progression. SSBP exemplifies this interaction, as genetic susceptibility and environmental sodium exposure converge to shape individual blood pressure responses [44].

Hypertension demonstrates significant variability among ethnicities and families, with strong heritability and a clear influence of family history. Although the majority of cases are polygenic, approximately 30% of cases arise from single-gene mutations that follow Mendelian inheritance patterns. These monogenic forms often present at a young age and may be associated with characteristic electrolyte abnormalities such as hypokalemia or metabolic alkalosis [45,46].

Several well-defined monogenic syndromes illustrate the link between sodium handling and salt sensitivity. For instance, Liddle’s syndrome—caused by gain-of-function mutations in the ENaC—leads to increased renal sodium reabsorption, volume expansion, and hypertension. Similarly, Gordon’s syndrome (also known as pseudohypoaldosteronism type II), involves mutations that upregulate NCC activity, resulting in salt-dependent blood pressure elevation. These disorders highlight how dysregulation of distal nephron sodium transport promotes salt-sensitive blood pressure responses [44]. A summary of key monogenic hypertensive disorders associated with salt sensitivity is provided in Table 1.

Beyond these rare syndromes, interindividual variability in renal sodium transport contributes substantially to SSBP in the general population. Genetic variation affecting key sodium transport pathways—including ENaC, NCC, and bicarbonate transporters such as SLC4A5—may amplify tubular sodium reabsorption and predispose patients to exaggerated blood pressure responses under high-salt conditions. Variants in taste receptors and sodium-sensing pathways have also been implicated, suggesting that both renal and extra-renal mechanisms influence sodium preference and handling [47,48].

While monogenic syndromes provide valuable insights, the majority of essential hypertension is polygenic, resulting from the cumulative effects of multiple common genetic variants. These include polymorphisms in genes regulating the RAAS, G-protein–coupled receptor (GPCR) signaling, and inflammatory pathways, such as interleukin-6 (IL-6 −572C/G), tumor necrosis factor-alpha (TNF-α −308G/A), and transforming growth factor-beta1 (TGF-β1) genes [44]. Among these, RAAS-related gene polymorphisms appear particularly relevant to salt sensitivity. For example, variants in angiotensinogen (AGT) (M235T, T174M) and the angiotensin-converting enzyme (ACE) gene (I/D polymorphism) modulate RAAS activation and influence individual BP responses to sodium intake. The ACE DD genotype, in particular, is associated with enhanced sodium retention and greater salt sensitivity, especially among men [44].

Experimental studies further reveal epigenetic mechanisms underlying salt-induced cardiovascular injury, with most data derived from experimental and translational models, whereas clinical interventional data in humans remain limited. High salt intake upregulates cardiac AGT and angiotensin II type 1 receptor (AT1R) expression, while mineralocorticoid receptor antagonists (MRAs) such as eplerenone attenuate this expression—independent of systemic BP reduction. Mechanistically, salt loading induces demethylation of the AGT promoter enabling recruitment of transcription factors such as CEBP and enhancing gene transcription. Mineralocorticoid receptor antagonism reverses this process by restoring methylation, suggesting a protective epigenetic effect and supporting the concept of tissue-specific RAAS epigenetic regulation as a therapeutic target [49,50].

In a broader context, epigenetic mechanisms—including DNA methylation, histone modification, and microRNA (miRNA) regulation—play a pivotal role in the development of salt sensitivity [51]. Hypomethylation of the AT1aR, AT1b, and ACE gene promoters has been associated with increased expression of these receptors and enzymes, ultimately contributing to elevated BP. Similarly, reduced methylation of TLR4 and IL-6 gene promoters enhances proinflammatory signaling, thereby promoting vascular dysfunction and hypertension.

Beyond DNA methylation, histone modifications also influence gene expression related to sodium handling and vascular tone. Experimental and translational studies have shown that alterations in histone marks—including increased H3K27me3 and acetylation of histones H3 and H4—modulate transcription of key renal sodium-handling genes such as WNK4 and the sodium–chloride cotransporter (NCC), both central to distal tubular sodium reabsorption and BP regulation.

In parallel, miRNA dysregulation has emerged as an additional epigenetic contributor. Alterations in miRNAs such as miR-126-3p, miR-182-5p, and miR-505 affect endothelial function, vascular inflammation, and overall BP homeostasis. These miRNAs are increasingly being explored not only as markers of disease activity but also as potential therapeutic targets in the management of salt-sensitive hypertension [13,44].

Finally, epigenetic alterations affecting vascular genes such as *CUL3* and *HSD11B2* can impair endothelial nitric oxide signaling and promote oxidative stress, leading to vascular stiffness and enhanced susceptibility to salt-induced organ damage. Unlike monogenic mutations, these epigenetic changes reflect dynamic regulatory disturbances that integrate environmental and dietary sodium exposure, thereby linking salt sensitivity to early cardiorenal dysfunction [52,53].

**Figure 2:**
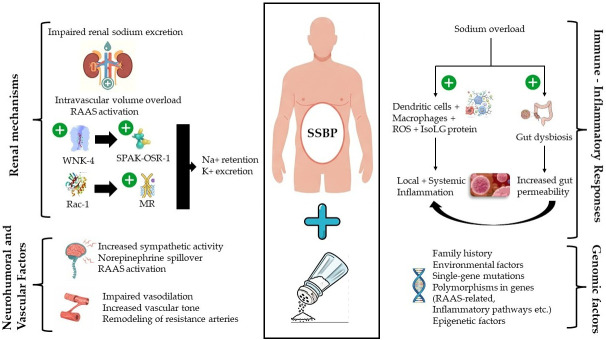
Integrated pathophysiological mechanisms underlying SSBP. The figure illustrates the converging renal, neurohormonal, vascular, immune–inflammatory, and genetic mechanisms contributing to the development of SSBP. Impaired renal sodium excretion leads to intravascular volume expansion and activation of the RAAS, promoting sodium retention and potassium excretion through pathways involving WNK4–SPAK/OSR1 signaling and Rac1-mediated MR activation. Ιncreased sympathetic nervous system activity, norepinephrine spillover, endothelial dysfunction, and vascular remodeling augment peripheral vascular resistance. Sodium overload also triggers immune and inflammatory responses, including activation of dendritic cells and macrophages, oxidative stress, IsoLG formation, and gut dysbiosis, further amplifying local and systemic inflammation. Genetic and epigenetic susceptibility modulates these processes, predisposing individuals to impaired sodium handling and exaggerated BP responses. Collectively, these interacting pathways establish a self-reinforcing cardiorenal–hypertensive phenotype characteristic of SSBP. Abbreviations: BP, blood pressure; IsoLG, isolevuglandins; MR, mineralocorticoid receptor; OSR1, oxidative stress–responsive kinase 1; Rac1, Ras-related C3 botulinum toxin substrate 1; RAAS, renin–angiotensin–aldosterone system; ROS, reactive oxygen species; SNS, sympathetic nervous system; SPAK, STE20/SPS1-related proline–alanine-rich kinase; SSBP, salt-sensitive blood pressure; WNK4, with-no-lysine (K) kinase 4.

Collectively, genetic and epigenetic data support biological plausibility for salt sensitivity as a cardiorenal phenotype; however, despite growing mechanistic insights, translation into routine clinical risk stratification and therapeutic decision-making remains limited [44,54]. Pathophysiological mechanisms underlying SSBP are depicted in Figure 2.

## 6. The Cardiorenal Continuum of Salt Sensitivity and Its Clinical Manifestations

### 6.1. Impact on Renal Microcirculation and GFR Decline

Building on the renal, immune and genetic mechanisms outlined above, salt sensitivity manifests clinically through progressive microvascular dysfunction and accelerated decline in renal function.

In salt-sensitive individuals, excessive sodium intake raises renal perfusion pressure and cortical vascular resistance, blunting the pressure–natriuresis relationship and disturbing tubuloglomerular feedback. This imbalance leads to medullary vasoconstriction, impaired perfusion of the vasa recta, tissue hypoxia and increased production of ROS. At the same time, tissue-level RAAS activation—regardless suppressed systemic levels—exacerbates afferent–efferent tone mismatch leading to glomerular hypertension and podocyte mechanical stress [55,56].

Early renal manifestations include glomerular hyperfiltration and microalbuminuria, which signal the onset of progressive GFR decline. This progression is mediated by podocyte damage, endothelial glycocalyx degradation, and mesangial expansion. Epigenetic mechanisms, AGΤ promoter demethylation and Rac1-mediated mineralocorticoid receptor activation, enhance tubular sodium reabsorption and interstitial inflammation, accelerating fibrotic remodeling and CKD progression [13,57,58].

Clinically, salt sensitivity may not initially present as persistent hypertension but rather as transient or modest BP elevations in response to sodium intake. Many patients exhibit a non-dipping nocturnal BP profile, reflecting impaired sodium handling and heightened sympathetic activity [59,60]. Salt-induced edema, subtle weight gain and early renal stress markers such as microalbuminuria or gradual rises in serum creatinine may precede overt disease [59,61]. In later stages, declining estimated glomerular filtration rate (eGFR) often coexists with left ventricular hypertrophy (LVH), persistent non-dipping patterns, and signs of systemic congestion [62].

Recognizing these early manifestations is crucial, as they often precede overt hypertension or renal dysfunction and identify patients who may benefit from early sodium restriction and targeted pharmacologic management [62]. From a therapeutic standpoint, dietary sodium restriction remains foundational, complemented by thiazide-like diuretics (e.g., chlorthalidone, indapamide), RAAS inhibition, sodium-glucose co-transporter 2 inhibitors (SGLT2i), and nonsteroidal MRAs to reduce glomerular pressure, limit albuminuria, and attenuate inflammatory renal injury [10,13,57].

### 6.2. Cardiac Remodelling and the HFpEF Connection

The salt-sensitive phenotype extends its impact beyond renal physiology, influencing myocardial structure and function through a combination of hemodynamic, neurohormonal, and inflammatory mechanisms. High dietary sodium intake expands plasma volume and increases systemic vascular resistance, thereby raising afterload and promoting concentric LVH. Chronic low-grade inflammation that characterizes salt-sensitive individuals further contributes to myocardial remodeling and impaired diastolic relaxation [8].

Endothelial dysfunction and oxidative stress interfere with the nitric oxide (NO)–cyclic GMP (cGMP)–protein kinase G (PKG) pathway, leading in titin hypophosphorylation, a protein helpful for maintaining heart muscle elasticity, consequently resulting in increased myocardial stiffness—biochemical hallmark of heart failure with preserved ejection fraction (HFpEF) [63,64]. Additionally, microvascular rarefaction, coronary endothelial inflammation, and extracellular matrix accumulation further reduce myocardial compliance. Collectively, this cascade results in left atrial myopathy and pulmonary venous hypertension, clinically manifesting as HFpEF phenotype, particularly in patients with concurrent CKD, diabetes mellitus, or obesity [64].

Clinically, these patients typically present with preserved ejection fraction, LVH and elevated E/e′ ratio on echocardiography, alongside mildly elevated natriuretic peptides, and salt-induced congestion. Diagnostic clues may include impaired exercise tolerance, exertional dyspnea, or post-prandial or evening symptoms likely related to volume redistribution [65,66].

Therapeutic management includes individualized sodium restriction, optimal blood pressure control—often with RAAS inhibitors and thiazide-like diuretics—and addition of MRAs and SGLT2i to reduce sodium-mediated fibrosis and improve ventricular compliance [67,68]. Beyond pharmacologic therapy, aggressive treatment of comorbidities—including obesity, metabolic syndrome, insulin resistance, obstructive sleep apnea, and sedentary lifestyle—is critical to prevent progression of HFpEF [69,70].

### 6.3. Interplay with Fibrotic and Inflammatory Pathways

Salt sensitivity bridges hemodynamic stress with chronic immune activation and fibrotic remodeling across the kidney–heart axis, accelerating both renal and cardiac disease progression. High extracellular sodium levels trigger immune dysregulation by promoting the formation of IsoLG–protein adducts, which activate antigen-presenting cells and the NLRP3 inflammasome, leading to Th17 cell polarization and IL-17A–mediated inflammation. These processes intensify oxidative stress and endothelial injury, laying the foundation for target organ damage [36,55]. In the kidney, Rac1-MR activation and TGF-β/SMAD signaling accelerate tubulointerstitial fibrosis, while in the myocardium, aldosterone/MR, Ang II/AT1R, galectin-3, and periostin pathways drive fibroblast proliferation and collagen deposition [71].

These maladaptive loops are further reinforced by local RAAS upregulation, oxidative stress, and epigenetic reprogramming, establishing a self-perpetuating cycle of sodium retention, microvascular dysfunction, and matrix expansion [72]. The inflammatory–fibrotic burden of SSBP is reflected by combined biomarker panels including albuminuria, NGAL, and cystatin C for renal injury, galectin-3, and soluble ST2 as indicators of cardiac fibrosis [59].

From a therapeutic standpoint, RAAS inhibitors, MRAs, SGLT2i, and targeted diuretic regimens form the cornerstone of treatment, helping to reduce glomerular pressure, mitigate inflammation, and slow fibrotic progression [73,74,75,76,77,78]. Emerging strategies such as IsoLG scavengers, inflammasome modulators, and interventions targeting the gut microbiome are currently under investigation and may offer novel therapeutic opportunities in salt-sensitive individuals [39,79,80].

## 7. Laboratory and Dynamic Testing in SSBP

**Figure 3:**
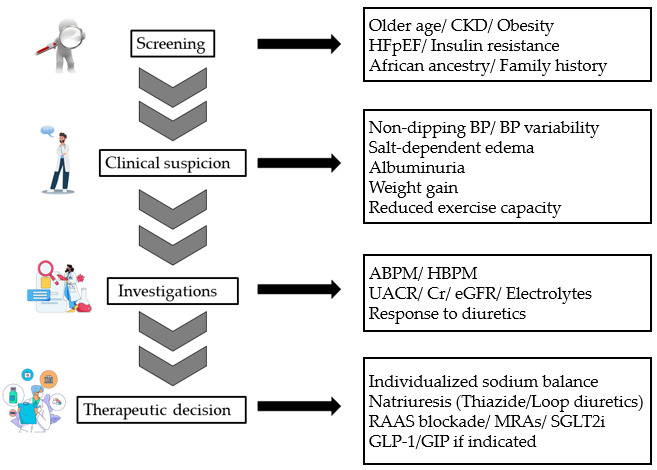
Clinical framework for the recognition and phenotype-guided management of SSBP. This schematic illustrates a stepwise, clinically oriented approach to SSBP, progressing from population-level screening and clinical suspicion to targeted investigations and individualized therapeutic decision-making. Risk factors prompting screening include older age, CKD, obesity, HFpEF, insulin resistance, African ancestry and family history. Clinical features raising suspicion include non-dipping or highly variable blood pressure patterns, salt-dependent edema, albuminuria, weight gain, and reduced exercise capacity. Diagnostic evaluation relies on dynamic blood pressure monitoring (ABPM/HBPM), laboratory assessment of renal function and electrolytes, and evaluation of natriuretic response. Management emphasizes individualized sodium balance and phenotype-guided therapy, including diuretics, RAAS blockade, mineralocorticoid receptor antagonists, SGLT2i, and incretin-based therapies when indicated. This framework reflects commonly applied clinical principles rather than a formal diagnostic algorithm. Abbreviations: ABPM, ambulatory blood pressure monitoring; BP, blood pressure; CKD, chronic kidney disease; Cr, serum creatinine; eGFR, estimated glomerular filtration rate; GLP-1, glucagon-like peptide-1 receptor agonist; GIP, glucose-dependent insulinotropic polypeptide; HBPM, home blood pressure monitoring; HFpEF, heart failure with preserved ejection fraction; MRA, mineralocorticoid receptor antagonist; RAAS, renin–angiotensin–aldosterone system; SGLT2i, sodium–glucose cotransporter-2 inhibitor; SSBP: salt-sensitive blood pressure; UACR, urine albumin-to-creatinine ratio.

Accurate identification and monitoring of SSBP require a combined approach of both dynamic assessment and laboratory testing. Together, these methods provide complementary information for hemodynamic behavior, renal and metabolic status, and treatment-related risks as well (Figure 3).

Traditional office BP measurements remain the most widespread tool for hypertension assessment, yet they inadequately capture the variability that salt-sensitive individuals demonstrate following sodium intake, volume expansion, and circadian shifts. Therefore, ambulatory blood pressure monitoring (ABPM), home BP monitoring (HBPM), and emerging wearable technologies provide more reliable estimates of true BP burden and its fluctuation. As emphasized by Schutte, Kollias, and Stergiou, dynamic BP metrics such as nighttime BP, dipping status, morning surge, and short-term variability are not only more reliable than office BP but also carry strong prognostic significance for cardiovascular outcomes in SSBP individuals [20].

Parallel to dynamic monitoring, laboratory evaluation plays a pivotal role in assessing sodium balance, detecting subclinical kidney injury, and planning antihypertensive therapy. Although no clinically validated tool can directly quantify whole-body sodium load, emerging techniques—such as bioimpedance spectroscopy for estimating total and extracellular body water and lung ultrasound for detecting early extravascular lung water—offer indirect insights into sodium-driven volume expansion, particularly in salt-sensitive phenotypes. Experimental methods such as ^23^Na-MRI, pioneered by Titze and colleagues, have demonstrated non-osmotic sodium storage in skin and muscle, but remain confined to research settings and are not applicable to routine clinical care [81,82,83]. Despite guideline recommendations, laboratory surveillance remains underutilized in clinical practice. Recent real-world data highlight that basic biochemical monitoring—including creatinine, eGFR, albuminuria, and serum electrolytes—is insufficiently applied, even among patients receiving RAAS inhibitors or diuretics. Notably, fewer than half of newly treated hypertensive patients undergo recommended lab evaluations in the first year of therapy [84,85,86]. This oversight may delay the recognition of hyperkalemia associated with RAAS blockade and MRAs, thiazide-related hyponatremia, acute kidney injury, progression of albuminuria related to salt-sensitive microvascular injury, and metabolic derangements such as dysglycemia—all of which contribute to worse cardiovascular and renal outcomes [87,88,89,90].

Hemodynamic consequences in salt-sensitive individuals are often mirrored by biochemical derangements. High dietary salt contributes to glomerular hyperfiltration, medullary microvascular hypoxia, and progressive eGFR decline, and early albuminuria signals endothelial glycocalyx injury and heightened intraglomerular pressure [91]. Electrolyte disturbances—particularly sodium, potassium, and chloride fluctuations—may arise spontaneously or be amplified by RAAS inhibitors, MRAs, or thiazide-type diuretics [92,93]. Additionally advanced renal biomarkers such as cystatin C, NGAL, and KIM-1 can detect early kidney stress before overt GFR loss, enhancing diagnostic sensitivity [94]. From a metabolic perspective, SSBP is often accompanied by insulin resistance and dyslipidemia, which further elevate cardiovascular risk [95].

**Figure 4:**
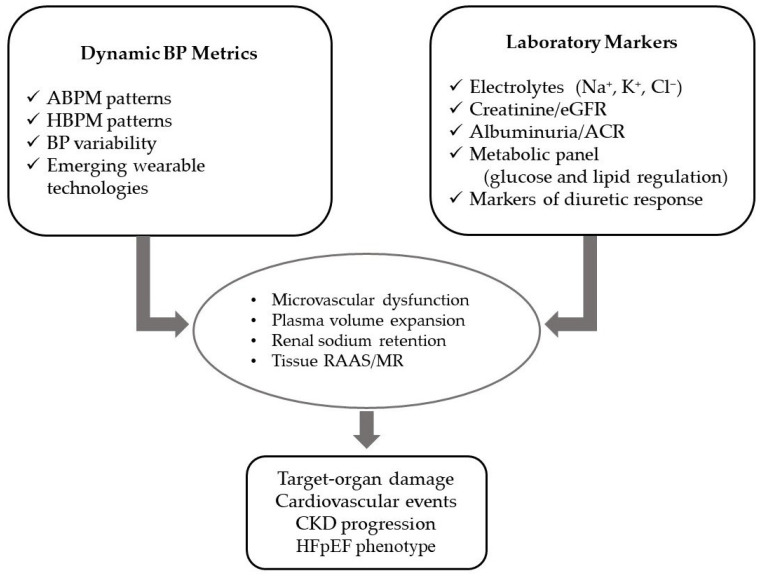
Framework of dynamic BP metrics and laboratory assessment in SSBP. The bidirectional correlation between dynamic hemodynamic evaluation and biochemical monitoring in the identification and management of SSBP. Dynamic BP indices, including ABPM and HBPM parameters, BP variability, and emerging wearable technologies measurements in combination with key laboratory markers relevant to SSBP pathophysiology, encompassing serum electrolytes (Na^+^, K^+^, Cl^−^), creatinine/eGFR, albuminuria/ACR, metabolic indicators, and markers of diuretic response target to early identification of hemodynamic and biochemical abnormalities (microvascular dysfunction, plasma volume expansion, renal sodium retention, and tissue RAAS/MR activation) that progressively result in major clinical consequences; target-organ damage, cardiovascular events, CKD progression, and the HFpEF phenotype. This integrative model provides a more complete risk profile, and facilitates precision diagnosis and individualized management in SSBP. Abbreviations: ABPM, ambulatory blood pressure monitoring; ACR, albumin-to-creatinine ratio; BP, blood pressure; CKD, chronic kidney disease; eGFR, estimated glomerular filtration rate; HBPM, home blood pressure monitoring; HFpEF, heart failure with preserved ejection fraction; MR, mineralocorticoid receptor; RAAS, renin–angiotensin–aldosterone system; SSBP: salt-sensitive blood pressure.

A standardized laboratory panel is essential for both initial workup and ongoing monitoring of SSBP patients. Key parameters should include serum electrolytes (Na^+^, K^+^, Cl^−^), creatinine and eGFR, urine albumin-to-creatinine ratio, fasting glucose and HbA1c, lipid profile, and markers of diuretic response such as urinary sodium. When combined with dynamic BP monitoring, these indices not only improve risk stratification but also support early detection of therapy-related complications, help tailor diuretic regimens, and guide timely intervention to prevent cardiorenal deterioration [96,97]. The schematic model of dynamic BP measurements and laboratory assessment in SSBP is described in Figure 4.

## 8. Therapeutic Strategies

### 8.1. Dietary and Lifestyle Interventions in SSBP

Dietary and lifestyle modification remains the cornerstone of managing SSBP, with the data strongly suggesting its capacity to lower BP, reduce cardiovascular risk, and enhance antihypertensive drug efficacy. Population-based data and recent guideline statements support dietary sodium reduction as a primary therapeutic target, with even modest reductions in sodium intake causing disproportionately larger decreases in BP considering salt-sensitive individuals. The Dietary Approaches to Stop Hypertension (DASH) diet—rich in fruits, vegetables, whole grains, potassium, and low-fat dairy—and Mediterranean diet have proven to be beneficial when combined with sodium restriction. These diets not only lower BP but also improve endothelial function, insulin sensitivity and lipid profiles. Conversely, limiting processed foods, saturated fats and added sugars contributes to better metabolic and vascular health.

Lifestyle modifications play an equally critical role. Weight loss and, regular aerobic exercise, reduce sympathetic overactivity and improve pressure–natriuresis relationships. A consistent physical activity schedule also helps reestablish circadian BP rhythms, promoting a healthy nocturnal dipping pattern. Additional strategies—such as moderating alcohol intake, smoking cessation, improving sleep hygiene, and addressing psychosocial stressors—are universally recommended by the International Society of Hypertension (ISH) and World Health Organization (WHO) to mitigate residual cardiovascular risk.

Collectively, these interventions alleviate the hemodynamic burden imposed by sodium excess and concurrently reduce inflammation, oxidative stress, and microvascular dysfunction—central contributors to the cardiorenal complications of SSBP [98,99,100].

### 8.2. Pharmacologic Management

The therapeutic goal in SSBP focuses on correcting sodium retention, blunting RAAS overactivity, and preventing downstream cardio–renal injury. Thiazide-like diuretics, such as chlorthalidone and indapamide, provide potent, sustained natriuresis and reduce blood pressure effectively in salt-sensitive individuals. While their traditional use is limited to patients with eGFR > 30–40 mL/min/1.73 m^2^, emerging data challenge this threshold. Notably, the CLICK trial demonstrated that chlorthalidone significantly lowered systolic BP in patients with stage 4 CKD (mean eGFR ~23) compared to placebo, supporting its role in volume-dependent, salt-driven hypertension, even at lower GFRs [78].

In more advanced CKD or overt volume overload, loop diuretics remain the preferred agents for volume and BP control. Importantly, diuretic efficacy may diminish when RAAS is activated—aldosterone upregulation enhances sodium reabsorption beyond the diuretic site of action, leading to a rightward shift in the pressure–natriuresis curve. Thus, concurrent RAAS blockade with ACE inhibitors or angiotensin receptor blockers (ARBs) is often required to restore natriuretic responsiveness. Observational studies confirm that higher RAAS activity is associated with poorer diuretic response, reinforcing this synergistic approach [101].

MRAs directly counter aldosterone-mediated sodium retention and interstitial fibrosis. Nonsteroidal MRAs such as finerenone offer antifibrotic benefits with a lower risk of hyperkalemia and may represent a safer long-term option in CKD and HF populations [102,103].

SGLT2i have demonstrated consistent cardiorenal benefit in large randomized clinical trials, including patients with CKD and HF phenotypes, supporting their clinical use beyond BP lowering. By inducing proximal tubular natriuresis, osmotic diuresis, and restoration of tubuloglomerular feedback, they result in modest plasma volume contraction (often reflected by rising hematocrit), reduced albuminuria, and urate excretion. Experimental models (e.g., Dahl salt-sensitive rats) demonstrate that SGLT2i lower BP on high-salt diets, modulate renal sodium transporters, and attenuate RAAS signaling—providing biological proof of concept for their role in SSBP [68,75].

Beyond classic ACEi/ARB therapy, next-generation RAAS-targeted strategies (e.g., angiotensin-(1–7)/Mas axis augmentation, aminopeptidase A inhibition, biased AT1R ligands) may offer future options to attenuate salt-driven vascular stiffness and fibrotic remodeling [102].

Glucagon-like peptide-1 receptor agonists (GLP-1 RAs) and dual glucose-dependent insulinotropic polypeptide (GIP)/GLP-1 agonists, they have shown favorable effects on weight reduction, metabolic inflammation, and functional capacity in selected populations, with growing evidence from randomized trials in obesity and HFpEF; however, their role in salt-sensitive hypertension remains indirect and phenotype-driven rather than BP-targeted [104,105].

More recently, AGT silencing through siRNA-based therapies has shown promising BP-lowering effects in early clinical trials (KARDIA-1 and KARDIA-2), introducing a long-acting, upstream RAAS-modulating approach that may be particularly relevant for salt-sensitive phenotypes. Current evidence is limited to early-phase clinical trials demonstrating BP reduction, while long-term cardiorenal outcomes and applicability to salt-sensitive phenotypes remain under investigation [106,107].

Targeting inflammasome-mediated pathways, particularly NLRP3, is promising yet largely experimental. It is supported primarily by preclinical and translational studies, while human observational data is limited and there is no definitive interventional clinical evidence to date [108].

Both pathophysiology and pharmacokinetics favor combination therapy for salt-sensitive phenotypes. Therapeutic choices include thiazide-like diuretic (or loop in overt volume overload) for natriuresis; ACEi/ARB to restrain RAAS and improve diuretic efficiency; MRA for aldosterone-linked sodium retention and antifibrosis; and SGLT2i for proximal-tubule/hemoconcentration effects and cardio-renal protection. This strategy is most compelling in patients with non-dipping BP patterns, albuminuria, edema, or CKD, where salt-sensitive mechanisms predominate [109].

### 8.3. Phenotype-Oriented Therapeutic Priorities in SSBP

#### 8.3.1. SSBP and Chronic Kidney Disease

In patients with SSBP and CKD, therapeutic priorities should focus on restoring sodium balance, attenuating neurohormonal activation, and limiting progressive renal injury. Core strategies include dietary sodium optimization, RAAS blockade, and agents with established nephroprotective effects, particularly SGLT2i and nonsteroidal MRAs. In individuals with concomitant obesity or type 2 diabetes, glucagon-like peptide-1 receptor agonists (GLP-1-RAs) may provide additional renal benefit by reducing albuminuria, attenuating metabolic and inflammatory burden, and slowing estimated glomerular filtration rate decline, thereby indirectly modulating salt-sensitive mechanisms without acting as primary antihypertensive agents. Recent randomized evidence supports the renal protective effects of GLP-1-RAs in patients with CKD and type 2 diabetes [33,110,111,112].

#### 8.3.2. SSBP and HFpEF

In SSBP associated with HFpEF, optimal regulation of BP and sodium balance represents a fundamental therapeutic priority. Effective BP control—achieved through dietary sodium optimization, RAAS inhibition, and appropriate diuretic strategies—is essential to limit volume overload, reduce ventricular afterload, and mitigate progressive myocardial and vascular remodeling. Within this framework, SGLT2i constitute a cornerstone of therapy, given their demonstrated benefits on HF hospitalization and cardiovascular outcomes and their Class I, Level A recommendation in contemporary European guidelines. Non-steroidal mineralocorticoid receptor antagonists further contribute by attenuating myocardial fibrosis and improving congestion control [113,114,115].

In HFpEF patients with elevated body mass index, GLP-1 RAs have emerged as a promising adjunctive strategy by improving exercise capacity, symptoms, and quality of life, while favorably modifying the cardiometabolic profile. More recently, dual GIP/GLP-1 receptor agonism has further expanded this therapeutic paradigm. In the SUMMIT trial, treatment with a GIP/GLP-1 RA was associated with significant weight reduction and improvements in functional capacity and patient-reported outcomes among individuals with obesity-related HFpEF. These effects, demonstrated in randomized clinical trials of obese HFpEF populations, may complement the natriuretic, hemodynamic, and antifibrotic actions of RAAS inhibition and SGLT2i in this salt-sensitive phenotype [105,116,117].

#### 8.3.3. SSBP and Obesity

In individuals with SSBP driven predominantly by obesity and insulin resistance, therapeutic strategies targeting proximal tubular sodium handling, metabolic inflammation, and sympathetic activation may offer disproportionate benefit. Alongside structured lifestyle intervention and SGLT2i, GLP-1 receptor agonists may further enhance phenotype-directed management through sustained weight loss, improvement of insulin sensitivity, and reduction of low-grade systemic inflammation, indirectly attenuating sodium retention and volume overload. Although incretin-based therapies are also relevant in HFpEF, their use in obesity-driven SSBP is primarily grounded in metabolic and renal mechanisms related to insulin resistance and sodium handling. Dual GIP/GLP-1 receptor agonism has demonstrated superior metabolic and weight-reducing effects compared with GLP-1 receptor agonism alone, with emerging evidence from obesity-related HFpEF populations supporting additional improvements in functional capacity and cardiometabolic burden. This phenotype-based framework underscores the heterogeneity of SSBP and supports individualized therapeutic prioritization rather than uniform BP targets [116,117,118,119].

**Figure 5:**
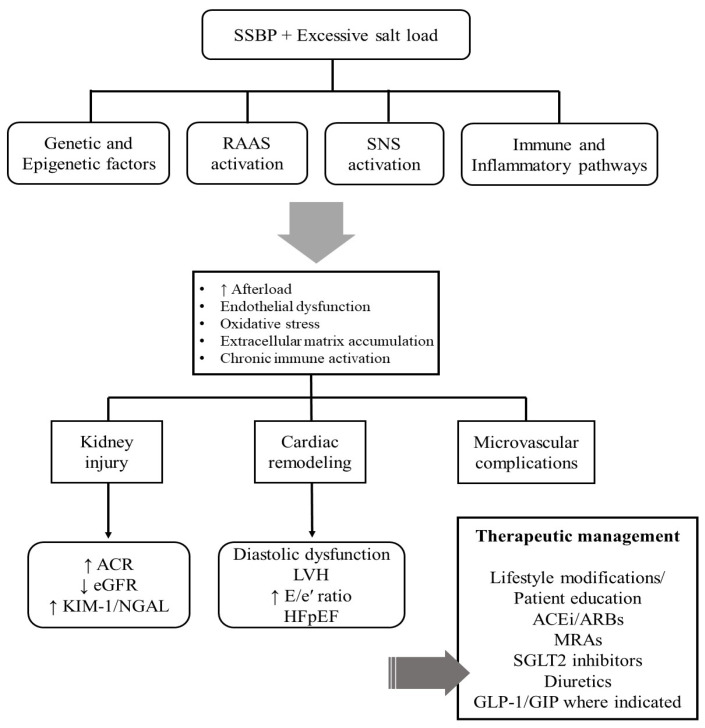
Pathophysiology, clinical manifestations and main therapeutic management of SSBP. Abbreviations: ACE, angiotensin-converting enzyme; ACR, albumin-to-creatinine ratio; ARBs, angiotensin receptor blockers; eGFR, estimated glomerular filtration rate; GIP, glucose-dependent insulinotropic polypeptide; GLP-1, glucagon-like peptide-1 receptor agonist; HFpEF, heart failure with preserved ejection fraction; LVH, left ventricular hypertrophy; MRAs, mineralocorticoid receptor antagonists; RAAS, renin–angiotensin–aldosterone system; SGLT2, sodium–glucose cotransporter 2; SNS, sympathetic nervous system; SSBP, salt-sensitive blood pressure.

The main pathophysiology, the clinical manifestations and cornerstones of therapeutic management are summarized in Figure 5.

### 8.4. Patient Education, Personalized Blood Pressure Targets and Therapeutic Escalation

Patient education represents a fundamental component of managing SSBP and should accompany pharmacological therapy from the earliest stages of care [120].

Given the dynamic and context-dependent nature of sodium-related BP variability, patients should be actively engaged in understanding dietary sodium sources, recognizing salt-induced symptoms such as edema or weight fluctuations, and adhering to prescribed treatment regimens. HBPM and periodic ambulatory BP assessment are particularly valuable in this population, as they allow detection of nocturnal non-dipping patterns, sodium-related BP surges, and treatment response beyond office measurements [12,33].

Personalized BP targets should be defined according to the individual clinical profile rather than uniform thresholds. Comorbidities such as CKD, HFpEF, diabetes mellitus, advanced age, and frailty influence both BP tolerance and therapeutic risk. In salt-sensitive individuals, individualized sodium balance—rather than indiscriminate sodium restriction—is essential to avoid counterproductive neurohormonal activation while preventing volume overload. Dynamic reassessment of BP, renal function, and electrolyte status is therefore critical to guide treatment intensity and ensure safety [14,72,121].

Effective implementation of these strategies increasingly relies on a multidisciplinary model of care integrating cardiology, nephrology, and advanced practice healthcare professionals. Multidisciplinary heart–kidney teams facilitate coordinated evaluation of volume status, optimization of diuretic and RAAS-targeted therapies, and early identification of treatment resistance. This collaborative approach is particularly relevant in patients with overlapping cardiometabolic disease, where therapeutic decisions must balance BP control, renal protection, and HF management [113,122,123].

Within this framework, structured patient education delivered by trained healthcare professionals—including advanced practice clinicians and specialized nursing staff—plays a pivotal role. These professionals contribute to dietary counseling, reinforcement of medication adherence, interpretation of home BP data, and early recognition of salt-induced decompensation. By supporting sustained behavioral change and timely therapeutic adjustment, team-based care enhances the translation of phenotype-guided strategies into real-world clinical practice [120,122,123].

When pharmacological treatment fails to achieve adequate BP control or clinical stability, systematic reassessment is required. This should include evaluation of adherence, dietary sodium intake, diuretic resistance, secondary contributors such as obstructive sleep apnea or drug interactions, and progression of renal dysfunction. Therapeutic escalation may involve optimization of diuretic strategy, intensification of renin–angiotensin–aldosterone system or mineralocorticoid receptor blockade, or referral to specialized hypertension or cardiorenal clinics. A structured, patient-centered, and multidisciplinary approach is essential to prevent ongoing cardiovascular and renal injury in refractory SSBP [124,125].

## 9. Perspectives for Clinical Practice and Translational Care

### 9.1. Clinical Recognition and Phenotype-Guided Management of SSBP

SSBP challenges the traditional “one-size-fits-all” approach to BP management by highlighting the heterogeneity of sodium handling, volume regulation, and cardiorenal vulnerability among hypertensive patients. Clinically, this phenotype often manifests through subtle but informative cues—non-dipping nocturnal BP patterns, salt-induced edema, weight gain, reduced exercise tolerance, early albuminuria, or disproportionate BP responses to dietary sodium—that frequently precede sustained hypertension or overt target-organ damage. The recognition of these patterns is essential for timely, phenotype-directed intervention [8,59,60,62,64].

Dynamic BP monitoring, including ambulatory and home-based measurements, plays a central role in identifying salt-sensitive hemodynamic behavior that may be missed by office readings alone. When combined with targeted laboratory assessment—such as serum creatinine, eGFR, UACR, urinary sodium excretion, and basic metabolic profiling—clinicians can better discern whether sodium-driven mechanisms predominate and tailor therapeutic intensity accordingly. In particular, the presence of elevated UACR and/or a declining eGFR should be interpreted not merely as markers of renal injury, but as signals of heightened salt sensitivity and cardiorenal risk, warranting intensified BP control and closer follow-up. From a nephrological perspective, persistent albuminuria or progressive reduction in eGFR despite standard therapy supports early optimization of RAAS inhibition and consideration of SGLT2i, which confer renal protection beyond BP lowering. In this context, laboratory abnormalities serve as actionable indicators guiding escalation of therapy rather than passive descriptors of disease severity [33,126].

Symptom assessment is equally important from a cardiological standpoint. Reduced exercise tolerance, peripheral edema, or salt-related weight fluctuations may indicate underlying diastolic dysfunction or evolving HFpEF. In such cases, timely cardiology evaluation and echocardiographic assessment are warranted, enabling early initiation of therapies targeting congestion, ventricular stiffness, and cardiometabolic contributors. Importantly, these manifestations often coexist with SSBP and may represent parallel expressions of the same pathophysiological substrate [127,128].

Obesity and insulin resistance further amplify salt sensitivity through effects on renal sodium reabsorption, sympathetic activation, and systemic inflammation. Routine assessment of body mass index, metabolic status, and lifestyle factors is therefore integral to clinical decision-making. Addressing obesity as a modifiable comorbidity—through structured lifestyle interventions and, when appropriate, pharmacologic strategies—can facilitate BP control, reduce volume burden, and lower long-term cardiovascular risk [105,116,117].

From a translational perspective, salt sensitivity provides a unifying framework linking hypertension, chronic kidney disease, and HFpEF across the cardiorenal continuum. Integrating dynamic BP metrics, laboratory markers of renal injury, symptom burden, and cardiometabolic profiling supports a shift toward phenotype-guided rather than uniform therapeutic strategies. Such an approach not only improves BP control but also targets the mechanisms most relevant to disease progression, fostering earlier intervention and more personalized care [129,130].

### 9.2. Concordance of Salt-Sensitive Blood Pressure with Current ESC/ESH Recommendations

Current guidelines from the European Society of Cardiology (ESC) and the European Society of Hypertension (ESH), as well as contemporary recommendations for CKD and HFpEF, emphasize individualized risk stratification, out-of-office BP assessment, and early organ protection rather than sole reliance on office BP thresholds. Within this framework, the concept of SSBP complements existing guideline principles by adding pathophysiological insight rather than representing a distinct or competing classification [131,132].

ESC/ESH guidelines acknowledge the heterogeneity of hypertension and recommend ABPM to detect masked hypertension, nocturnal hypertension, and non-dipping patterns—phenotypes frequently associated with impaired sodium handling. In this context, SSBP aligns closely with guideline-endorsed BP phenotyping [131,132].

Similarly, CKD guidelines recognize albuminuria, volume status, and RAAS activation as key determinants of disease progression and cardiovascular risk, independent of eGFR. The SSBP framework integrates these elements by linking impaired natriuresis, microvascular injury, and neurohormonal activation into a unified phenotype that may precede overt renal dysfunction, providing a mechanistic rationale for prioritizing sodium restriction and RAAS blockade in selected patients [131,133].

In HFpEF, consensus documents emphasize volume sensitivity, arterial stiffness, metabolic inflammation, and comorbidity burden—features that substantially overlap with salt-sensitive phenotypes, particularly in patients with obesity, CKD, and non-dipping or nocturnal BP patterns. Although salt sensitivity is not explicitly defined in HFpEF guidelines, recommended therapies such as diuretics, RAAS modulation, SGLT2 inhibitors, and sodium restriction are consistent with the underlying pathophysiology of SSBP [8,134].

Notably, current guidelines do not formally operationalize salt sensitivity as a diagnostic entity, reflecting the absence of standardized testing and outcome-driven evidence. Accordingly, SSBP should be viewed as a complementary, phenotype-based construct that may enhance early risk identification and support personalized therapeutic decisions within existing guideline frameworks [131,132].

## 10. Limitations

This review has limitations inherent to its narrative design. The absence of a systematic methodology and formal risk-of-bias assessment may introduce selection bias. Heterogeneity in definitions and diagnostic protocols for salt sensitivity limits direct comparison across studies. Furthermore, many mechanistic insights derive from experimental or observational data, which may not fully translate into routine clinical practice. These limitations underscore the need for standardized diagnostic tools and prospective studies.

## 11. Conclusions and Future Directions

Salt-sensitive hypertension represents a clinically meaningful yet underrecognized phenotype at the intersection of cardiovascular and renal disease. Beyond dietary sodium intake alone, it reflects impaired pressure–natriuresis, neurohormonal activation, immune-mediated inflammation, and progressive fibrotic remodeling across the heart–kidney axis.

From a clinical perspective, recognizing salt sensitivity as an early cardiorenal phenotype has direct implications for risk stratification and management. Dynamic BP assessment, targeted laboratory evaluation, and attention to volume- and sodium-related cues may allow the identification of high-risk individuals before irreversible structural organ damage develops. In this context, individualized sodium balance, adequate potassium intake, and mechanism-oriented pharmacological strategies—including thiazide-like diuretics, RAAS inhibition, MRAs, and SGLT2i—remain the cornerstone of current management. Looking forward, the management of SSBP lies in precision medicine. Future research should focus on validating pragmatic diagnostic algorithms that combine BP phenotypes with mechanistically informed biomarkers. Emerging approaches—such as genomic and epigenetic profiling, immune and inflammatory pathway modulation, and digital health tools including wearable BP monitoring and sodium-tracking technologies—may further refine early detection and enable personalized therapeutic targets. Ultimately, reframing SSBP as an early manifestation of cardiorenal vulnerability shifts the clinical focus to early disease modification, aiming to alter long-term cardiovascular and renal trajectories.

## Figures and Tables

**Table 1:** Gene mutations responsible for monogenic hypertension and their clinical manifestations.

Disease	Gene Mutations and Results	Clinical Manifestations
Apparent Mineralocorticoid Excess (AME)	*HSD11B2* gene → 11β-HSD2 enzyme activity deficiency (>40 mutations identified)	Cortisol-induced mineralocorticoid receptor activation Sodium retention Hypertension
Liddle’s Syndrome (LS)	*SCNN1B* or *SCNN1G* gene (autosomal dominant) → ENaC dysfunction → hyperactivity and increased sodium reabsorption	Low renin/aldosterone hypertension (responsive to amiloride)
Glucocorticoid-Remediable Aldosteronism (GRA)	*CYP11B1* and *CYP11B2* chimeric gene → ACTH-dependent aldosterone overproduction	Early-onset hypertension (treatment with glucocorticoids)
Gordon’s Syndrome (GS)	*WNK1, WNK4, CUL3*, or *KLHL3*→ thiazide-sensitive NCC dysregulation	Hyperkalemia Metabolic acidosis Thiazide-responsive hypertension

Abbreviations: 11β-HSD2, 11β-hydroxysteroid dehydrogenase type 2; ACTH, adrenocorticotropic hormone; CUL3, cullin 3; CYP11B1, cytochrome P450 family 11 subfamily B member 1; CYP11B2, cytochrome P450 family 11 subfamily B member 2; ENaC, epithelial sodium channel; HSD11B2, 11β-hydroxysteroid dehydrogenase type 2; KLHL3, kelch-like protein 3; NCC, sodium–chloride cotransporter; SCNN1B, sodium channel non–voltage-gated 1 beta subunit; SCNN1G, sodium channel non–voltage-gated 1 gamma subunit; WNK1, with no lysine (K) kinase 1; WNK4, with no lysine (K) kinase 4.

## Data Availability

No new data were created or analyzed in this study. Data sharing is not applicable to this article.

## Abbreviations

The following abbreviations are used in this manuscript:

11β-HSD2	11β-Hydroxysteroid Dehydrogenase Type 2
ABPM	Ambulatory Blood Pressure Monitoring
ACE	Angiotensin-Converting Enzyme
ACEi	Angiotensin-Converting Enzyme Inhibitor
ACTH	Adrenocorticotropic Hormone
ACR	Albumin-Creatinine Ratio
AGT	Angiotensinogen
ARBs	Angiotensin Receptor Blockers
AT1R	Angiotensin II Type 1 Receptor
BP	Blood Pressure
cGMP	Cyclic Guanosine Monophosphate
CKD	Chronic Kidney Disease
CUL3	Cullin 3
CEBP	CCAAT-Enhancer Binding Protein
DASH	Dietary Approaches to Stop Hypertension
eGFR	Estimated Glomerular Filtration Rate
ENaC	Epithelial Sodium Channel
ESC	European Society of Cardiology
ESH	European Society of Hypertension
FOXO1	Forkhead Box protein O1
GFR	Glomerular Filtration Rate
GIP	Glucose-dependent insulinotropic polypeptide
GLP-1 RA	Glucagon-like peptide-1 receptor agonist
GPCR	G-Protein–Coupled Receptor
HF	Heart Failure
HFpEF	Heart Failure with Preserved Ejection Fraction
HBPM	Home Blood Pressure Monitoring
HSD11B2	11β-Hydroxysteroid Dehydrogenase Type 2 Gene
IL-1β	Interleukin-1 Beta
IL-6	Interleukin-6
IL-10	Interleukin-10
IL-17A	Interleukin-17A
ISH	International Society of Hypertension
IsoLG	Isolevuglandins
KDIGO	Kidney Disease: Improving Global Outcomes
KIM-1	Kidney Injury Molecule-1
KLHL3	Kelch-Like Protein 3
LVH	Left Ventricular Hypertrophy
M1/M2	Macrophage Subtypes 1 and 2
MAP	Mean Arterial Pressure
miRNA	MicroRNA
MR	Mineralocorticoid Receptor
MRAs	Mineralocorticoid Receptor Antagonists
NCC	Sodium-Chloride Cotransporter
NGAL	Neutrophil Gelatinase-Associated Lipocalin
NO	Nitric Oxide
NLRP3	NOD-Like Receptor Pyrin Domain-Containing 3
PKG	Protein Kinase G
RAAS	Renin–Angiotensin–Aldosterone System
Rac1	Ras-Related C3 Botulinum Toxin Substrate 1
ROS	Reactive Oxygen Species
SCNN1B	Sodium Channel Non-Voltage-Gated 1Beta Subunit
SCNN1G	Sodium Channel Non-Voltage-Gated 1 Gamma Subunit
SGK1	Serum and Glucocorticoid-Regulated Kinase 1
SGLT2i	Sodium-Glucose Co-Transporter 2 inhibitors
SSBP	Salt-Sensitive Blood Pressure
SNS	Sympathetic Nervous System
ST2	Suppression of Tumorigenicity 2 (soluble)
TGF-β	Transforming Growth Factor-Beta
Th17	T Helper 17 Cells
TLR4	Toll-Like Receptor 4
TGF-β1 (appears as β1)	Transforming Growth Factor-Beta 1
TNF-α	Tumor Necrosis Factor-Alpha
TonEBP	Tonicity-Responsive Enhancer Binding Protein
VEGF-C	Vascular Endothelial Growth Factor C
WNK1	With-No-Lysine (K) Kinase 1
WNK4	With-No-Lysine (K) Kinase 4
